# The effect of postpartum vitamin A supplementation on breast milk immune regulators and infant immune functions: study protocol of a randomized, controlled trial

**DOI:** 10.1186/s13063-015-0654-9

**Published:** 2015-03-31

**Authors:** Shaikh Meshbahuddin Ahmad, Md Iqbal Hossain, Peter Bergman, Yearul Kabir, Rubhana Raqib

**Affiliations:** Center for Vaccine Sciences, icddr,b, Mohakhali, Dhaka, 1212 Bangladesh; Center for Nutrition and Food Security, icddr,b, Mohakhali, Dhaka, 1212 Bangladesh; Karolinska University Hospital, Huddinge, S-141 86 Stockholm, Sweden; Department of Biochemistry and Molecular Biology, University of Dhaka, Dhaka, 1000 Bangladesh

**Keywords:** Postpartum vitamin A supplementation, Infant immunity, Randomized controlled clinical trial

## Abstract

**Background:**

Because of limited impact on infant morbidity, mortality, and vitamin A status, the new guideline of the World Health Organization (WHO) does not recommend postpartum vitamin A supplementation (VAS) as a public health intervention in developing countries. However, breast milk contains numerous immune-protective components that are important for infant immune development, and several of these components are regulated by vitamin A.

**Methods/design:**

Postpartum women are being enrolled within 3 days (d) of delivery at a maternity clinic located in a slum area of Dhaka city and randomized to one of four postpartum VAS regimens (32/group, total 128). The regimens are as follows: Group 1: 200,000 IU VAS at <3 d and placebo at 6 weeks postpartum; Group 2: placebo at <3 d and 200,000 IU VAS at 6 weeks postpartum; Group 3: 200,000 IU VAS, both at <3 d and 6 weeks postpartum; Group 4: placebo, both at <3 d and 6 weeks postpartum. Breast milk samples at <3 d (before supplementation) and 4 months postpartum will be used to measure vitamin A and bioactive compounds. Infant blood samples at 2 and 4 months of age will be used to measure vitamin A, as well as innate and vaccine-specific immune responses. Dietary, anthropometric, and morbidity data are also being collected.

**Discussion:**

This is the first placebo-controlled randomized clinical trial of postnatal vitamin A supplementation to investigate the key bioactive compounds in breast milk, important for infant immunity, in relation to dose and time point of postpartum supplementation and whether such maternal supplementation improves infant immune status during the critical period of early infancy.

**Trial registration:**

ClinicalTrials.gov: NCT02043223, 5 December 2013

## Background

Vitamin A deficiency increases the risk of death from infections in children and is still a major public health problem in developing countries. At present, vitamin A supplementation (VAS) is recommended for children beginning at 6 months of age to reduce vitamin A deficiency and risk of death from infection. Up to age 6 months, breast milk is the only source of vitamin A for infants. However, there is ample evidence that, in addition to essential nutrients, breast milk contains numerous immune-protective components [1,2]. Proteomic analysis reveals 268 proteins in human milk, of which 44 are related to host defense, mostly involved in the immune system [3], and several of them are regulated by vitamin A [4-7]. However, studies on the effect of postpartum VAS on breast milk immune regulators are extremely limited. One study reported higher secretory IgA levels in breast milk after immediate postpartum VAS [8]. Another study demonstrated no effect on milk concentrations of immune factors including secretory IgA and IL-8 at 3 months after postpartum VAS within 1–3 weeks of delivery [9]. These studies lack follow-up data and data on other key immune regulators in breast milk or the effect of maternal VAS on infant immune development. Nevertheless, it is well characterized that the immunological composition of human milk changes over the lactation period [2,10]. To increase mothers’ vitamin A stores in developing countries, which are depleted during the course of pregnancy and lactation, and to increase the vitamin A content in breast milk, in 1997 the WHO/UNICEF/IVACG Task Force recommended 200,000 IU VAS of postpartum women within 6 weeks of delivery [11]. Because of limited impact on the infant vitamin A status, those guidelines were revised in 2001, and 400,000 IU VAS, divided into two equal doses, were recommended at least one day apart [12]. Systematic reviews found no impact of postpartum VAS on maternal and infant morbidity and mortality [13-15], and the new 2011 WHO guideline did not recommend postpartum VAS as a public health intervention [16].

However, in several studies mothers were supplemented with vitamin A within 6 weeks of delivery, without stratifying the supplementation time point during the postpartum period. This is a major limitation in studying the clinical impact of maternal supplementation, because colostrum (produced the first few days postpartum) is three times richer in vitamin A and 10 times richer in beta-carotene (provitamin A) than mature milk (produced 2 weeks after delivery). High-dose VAS failed to increase colostrum vitamin A levels in mothers with lower serum retinol levels [17]. Thus, vitamin A metabolism, immune-regulatory effect, and transfer to mammary gland differ if supplementation is carried out in the immediate or late postpartum period in regions where the deficiency is endemic. The primary objective of this study is to determine if postpartum VAS enhances immune components in breast milk and whether this effect is modified by the dosing regimen during the postpartum period (immediate or delayed). Secondary, exploratory objectives include the effect of postpartum VAS on infant immune status by determining microbial pattern recognition receptor-mediated innate immune responses, T cell responses, and vaccine-specific antibody-secreting plasma cell responses.

## Methods/design

### Study approval

The study protocol was peer reviewed and approved by the Research Review Committee (RRC) and Ethical Review Committee (ERC) of the International Centre for Diarrhoeal Disease Research, Bangladesh (icddr,b) (FWA-00001468; IRB-00001822).

### Study site and population

This study is being carried out at icddr,b, Dhaka, in collaboration with the Maternal and Child Health Training Institute (MCHTI) at Azimpur. Study participants live in the largest concentration of slums, located at the western fringe, having an urban slum population of about 1 million. MCHTI is the largest public maternity clinic located in that area. This clinic provides antenatal and prenatal care at minimal or no cost. According to the clinic registry, there are about 400 babies born per month. We are obtaining informed written consent from the pregnant women. During the immediate postpartum period, we also renew the consent of the mother and her willingness to have her infant participate.

### Study design

To test the impact on immune components of breast milk and infant immune function, we are carrying out a prospective, double-masked, randomized controlled clinical trial of postpartum vitamin A supplementation (VAS).

#### Inclusion/exclusion criteria

The inclusion criteria for postpartum women are as follows: (1) lives nearby MCHTI clinic, for convenience of follow-up visits; (2) ≥18 years of age; (3) free from diabetes, neoplasia, or other serious chronic or infectious diseases; (4) has given birth to a singleton baby; and (5) willingness to have the infant participate and adhere to the protocol. Inclusion criteria for newborn babies are: (1) free from congenital disease or a serious infection and (2) eligible for vaccination according to the MCHTI clinic policy.

#### Randomization and study intervention

**Figure 1:**
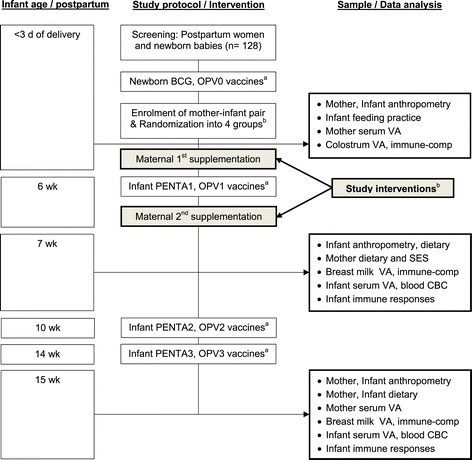
**Study flow chart.** ^a^According to the national Expanded Program for Immunization (EPI) for newborn and infants. OPV, oral poliovirus vaccine; PENTA, pentavalent vaccines- a combination of five vaccines that comprise diphtheria, tetanus, whole cell pertussis, hepatitis B and haemophilus influenzae type b conjugate. ^b^Based on maternal supplementations **(Study interventions): Group 1:** 200,000 IU VA at <3 d and placebo at 6 wk; **Group 2:** placebo at <3 d and 200,000 IU VA at 6-wk; **Group 3:** 200,000 IU VA, both at <3 d and 6 wk; and **Group 4:** placebo, both at <3 d and 6 wk.

After obtaining informed written consent, eligible postpartum women within 2 to 3 days of delivery are being block-randomized with a block size of 4 to achieve equal allocation ratios across groups. A computer-generated, randomized sequence within a block is utilized to assign women into the following four groups (n = 32/group, total 128) for the supplementation of 200,000 IU vitamin A and/or a placebo during immediate (<3 days, d) and/or delayed (6 weeks) postpartum periods (Figure 1).

- Group 1: 200,000 IU vitamin A at <3 d and placebo at 6 weeks postpartum;
- Group 2: placebo at <3 d and 200,000 IU vitamin A at 6 weeks postpartum;
- Group 3: 200,000 IU vitamin A, both at <3 d and 6 weeks postpartum; and
- Group 4: placebo, both at <3 d and 6 weeks postpartum.

#### Safety considerations

We do not anticipate any serious adverse events as a result of once or twice supplementing with 200,000 IU vitamin A at 6 weeks apart in a community where the prevalence of low serum retinol (<1.05 μmoI/L) among lactating mothers is >26% with approximately 10% of them classified as deficient (serum retinol <0.70 μmol/L) and dietary sources of vitamin A are also limited (unpublished data). Earlier, similar doses of postpartum VAS were recommended by the WHO/UNICEF/IVACG Task Force [11,12]. Large doses of vitamin A are well tolerated by newly delivered mothers [18]. However, typical symptoms of acute hypervitaminosis A in adults include headache (presumably from increased intracranial pressure), nausea, vomiting, and occasionally fever and visual disorientation. These symptoms are generally transient and do not lead to permanent adverse effects [19]. It is also unlikely to have chronic vitamin A toxicity among study participants (symptoms are, for example, anorexia, dry itchy skin, osteoporosis, and hip fracture). These conditions can result from daily intakes of >25,000 IU vitamin A for >6 years or >100,000 IU vitamin A for >6 months [19-21]; nevertheless, there is wide inter-individual variability for the lowest intake required to elicit such toxicity. In addition, postpartum VAS shows no overall effect on disease transmission, for example, maternal-to-child transmission of HIV [22].

#### Sample size calculation

In this study, our primary aim is to compare the mean responses of immune markers in breast milk following postpartum VAS compared with a placebo. There are limited data available with human studies. In one study, supplementing postpartum women with a single 200,000 IU vitamin A dose increased secretory immunoglobulin A (sIgA) concentration in breast milk by 45.7% compared to a placebo within one day [8]. We are interested in determining if at least 20% higher immune markers (including sIgA) in breast milk persist at 4 months among women who receive a single dose of 200,000 IU vitamin A either at <3 d (Group 1) or at 6 weeks (Group 2) postpartum compared to the placebo (Group 4) and 10% higher responses among women who receive two doses of 200,000 IU vitamin A both at <3 d and 6 weeks (Group 3) postpartum compared to single-dosed postpartum women. In a one-way ANOVA design, sample sizes of 29 in each of the four groups achieves 80% power to detect our predicted differences using an *F*-test with 0.05 significance levels. The size of the variation in the means is represented by a standard deviation (SD) of 39.5 mg/dL of sIgA. The common SD within a group is assumed to be 126 mg/dL, a pooled value obtained from a previous trial [8]. There are no data available regarding immune measures of infants resulting from maternal VAS. In this project we consider that lymphocyte antibody responses in infants of the four maternal groups are the prime outcome variables which might link to the protection of infants from infection. With 29 infants per group, we will have >80% statistical power to detect a mean difference of 75% of the population standard deviation at α = 0.05 based on direct comparison between infants of the placebo group and infants of any of the three maternal VAS groups. Considering 10% attrition, our goal is to recruit 32 postpartum women into each of the four groups, for a total of 128 participants.

#### Sample/data collection

Breast milk sample collection is standardized to reduce bias and potential diurnal variability. Hindmilk is chosen to make a uniform sample across participants, since the constituents of milk change from the start to the end of a feeding. Mothers are being asked to provide 3–5 mL of milk samples that flowed towards the end of a feeding or a breast pumping session between 8 and 9 A.M. Milk samples are being collected in sterile 15-mL plastic tubes and immediately placed in an ice box and transported to the icddr,b laboratory within 4 hours of collection, where they are stored at −80°C in 500-μL aliquots until analysis. Breast milk samples are being collected at <3 d (before supplementation), 7 weeks, and 15 weeks postpartum. Blood samples of 2–3 mL from infants and mothers are being collected at 7 weeks and 15 weeks (Figure 1). A clinic nurse obtains blood samples in heparinized tubes.

During follow-up, field workers use structured questionnaires in paper form to collect data on pregnancy history, anthropometry, demography, diet, morbidity, and time/date of maternal supplementation, infant vaccination, and biological sample collection (Figure 1).

### Laboratory investigations

#### Assessment of breast milk immune components

Breast milk contains an array of bioactive compounds. In this study, we select key immune markers that represent overall functional competence of breast milk and rationalize with infant immune responses. In the anti-microbial and microbial growth regulator categories, we measure secretory immunoglobulin A (sIgA) and soluble cluster of differentiation antigen 14 (sCD14), respectively. In addition to non-specific opsonization of pathogens for phagocytosis and destruction, breast milk sIgA has DNase- and RNase-like activity [23] that could protect infants by hydrolyzing pathogenic viral and bacterial nucleic acids [24]. CD14 participates in inflammatory responses against the lipopolysaccharide (LPS) of Gram-negative infections [25] and also recognizes soluble peptidylglycan from the Gram-positive bacterial cell wall [26]. In the tolerance-priming and anti-inflammatory category, we measure transforming growth factor beta (TGF)-β [27], which specifically enhances IgA production [28] and promotes healing of intestinal cells damaged by infection [29]. In the lymphopoietic and colony-stimulating categories, we will measure interleukin (IL)-7 and granulocyte-monocyte colony-stimulating factor (GM-CSF), respectively. IL-7 is involved in peripheral homeostasis of all major T cell subsets, including naïve [30] and memory [31] cells. GM-CSF is characterized by its ability to stimulate neutrophil, monocyte/macrophage, and eosinophil colony formation. It can also enhance phagocytosis and antibody-dependent cell-mediated cytotoxicity [32].

#### Experimental procedure

Frozen milk samples will be thawed at 37°C and centrifuged at 13,000 rpm for 15 min at 4°C. The upper fatty layer will be removed, and the middle aqueous layer will be extracted by pipette for the measurement of sIgA, sCD14, IL-7, and GM-CSF by enzyme-linked immunosorbent assay (ELISA) kits [33]. Since TGF-β is bound in the fat portion of the milk [34], milk samples will be acidified by the addition of 1 N HCl for 10 min at room temperature followed by neutralization by 1.2 N NaOH/0.5 M HEPES, as described earlier [33].

### Assessment of infant immune functions

#### Innate immune responses

The innate immune system provides the first line of defense against invading microbes. Activation of pattern recognition receptors, such as Toll-like receptors (TLRs), in immune cells leads to the release of immune regulators which have direct antimicrobial activity and can also shape the adaptive immune system. In this study, we assess the cytokine responses to TLR4 and TLR9 stimulation. TLR4 expression on monocytes and neutrophils identifies bacterial LPS, a major outer wall structural component of Gram-negative bacteria. We evaluate inflammatory status by measuring tumor necrosis factor (TNF)-α/IL-10 responses to LPS stimulation. TLR9, preferentially expressed on plasmacytoid dendritic cells, recognizes microbial unmethylated CpG oligodeoxynucleotides (CpG ODN) sequences. We measure type 1 interferon (IFN)-α in response to CpG ODN stimulation. IFN-α plays a key role against virus replication [35] and induces the expansion of cytotoxic T cells [36,37].

#### Experimental procedure

To study innate immunity, 100 uL whole blood (1:1 in 10% autologous plasma containing RPMI-1640) is cultured for 24 hours at 37°C and 5% CO_2_ with TLR4 agonists MPLA (monophosphoryl lipid A, Invivogen)/LPS (Sigma) at 1.0 ug/mL and with TLR9 agonist ODN-2216 (Type A CpG oligonucleotide, Invivogen) at 1 uM. Optimum concentrations of these agonists for culture have been selected from our previous experiments and other studies [6,38,39]. After incubation, cell-free supernatants are collected and stored at -80° with 1% protease inhibitor for the analysis of TNF-α and IL-10 (MPLA/LPS culture) and IFN-α (CpG ODN culture) by ELISA kits.

#### T cell polarization responses

Appropriate adaptive immune responses are required for recovery from normal, childhood infections. For the assessment of adaptive immune competence, we will measure distinct differentiation of T helper (Th) cells into four different subtypes based on expression of signature cytokine in response to mitogenic stimulation. Signature cytokines for Th1, Th2, Th17, and T regulatory (Tr)-1 are IFN-γ, IL-13, IL-17, and IL-10, respectively. The Th1 response promotes defense against intracellular pathogens, while the Th2 response promotes defense against extracellular pathogens and allergic reaction. Th17 cells play important roles in various autoimmune conditions and the progression of tumors. Tr1 cells are an inducible subset of regulatory T cells that play a pivotal role in promoting and maintaining tolerance [40].

#### Experimental procedure

To study epitope-independent T cell mitogen mediated cytokine responses, diluted (1:4) whole blood is cultured with 2 ug/mL SEB (Staphylococcal enterotoxin B, Sigma) for 3 days. After incubation, cell-free supernatants are collected and stored at -80^0^ with 1% protease inhibitor for the analysis of IFN-γ, IL-13, IL-17, and IL-10 by ELISA kits.

#### Vaccine responses

We utilize a novel cell-mediated method for measuring the vaccine-specific antibody response from mononuclear cells termed antibody in lymphocyte supernatant (ALS) culture [41]. Although serum antibody is widely used to assess post-vaccination immunity, it is not possible to distinguish between recently produced antibodies and pre-existing antibodies as a result of previous antigen exposure or from passively derived maternal antibodies. Serum antibodies include the accumulated soluble antibodies, while the ALS assay quantifies only the amount and strength of circulating, antibody-secreting plasma cells from a recent immunization. To obtain these plasma cells, we collect infant blood samples 1 week after the first and 1 week after the third doses of vaccination (Figure 1). After vaccination, antibody-secreting cells in the draining lymph nodes transiently peak and then decline within a few weeks due to selection for higher affinity plasma cells and apoptotic loss of low-affinity cells, and at the same time cells begin to migrate and accumulate in the bone marrow compartment, which remains the predominant site of antibody production [42,43]. Because of the methodological difficulties involved in investigating bone marrow plasma cells, we use the ALS method as described in our previous study [5].

#### Experimental procedure

To study the extent of vaccine-specific antibody-secreting plasma cell generation following single and three doses of immunization, we separate peripheral blood mononuclear cells (PBMCs) using the Ficoll density gradient procedure, and 1 x 10^7^ cells/mL in RPMI-1640 with 10% fetal bovine serum (FBS) are cultured without any stimulation for 3 days at 37°C and 5% CO_2_. Cell-free supernatants are then harvested to determine pertussis toxin and hepatitis B surface antigen-specific IgG responses by commercially available ELISA kits.

#### Assessment of vitamin A status

The vitamin A levels of breast milk and mother and infant plasma will be measured by HPLC [44]. Since vitamin A is associated with milk fat, which varies within the feeding episode, breast milk vitamin A content will be expressed as concentration per gram of milk fat, measured by the creamatocrit method. These methods are now currently in place at icddr,b [45].

### Outcome variables

The primary outcome variables are breast milk immune regulators at <3 d (before supplementation), 7 weeks, and 15 weeks postpartum. These variables are:

- sIgA, sCD14, TGF-β, IL-7, and GM-CSF, and
- Vitamin A status

The secondary outcome variables are associated with infant immune responses at 7 weeks and 15 weeks of age as a result of maternal postpartum VAS. These variables are:

- Microbial pattern recognition receptors (Toll-like receptor, TLR) and stimulated TNF-α, IL-10, and IFN-α responses,
- Epitope-independent mitogen (PHA) stimulated T cell polarization: IFN-γ (Th1), IL-13 (Th2), IL-17 (Th17), and IL-10 (Tr1),
- Pertussis toxin and hepatitis B surface antigen-specific IgG antibody-secreting plasma cell responses after single dose (at 7 weeks) and multiple doses (15 weeks) of vaccinations, and
- Plasma vitamin A.

Other variables include mother’s plasma vitamin A, infant complete blood count (CBC) including hemoglobin and other red blood cell indices, and infant anthropometry and morbidity data.

### Data management

Data obtained from the field site are being entered into a predesigned database created by using the Epi Info 7 program (developed by the Centers for Disease Control, CDC) for this project. 20% data is checked by independent verification. Laboratory data generated from ELISA are exported directly from the instruments into Excel-compatible data files, while other laboratory data, such as CBC and retinol data, are entered into Excel spreadsheets. All these data will be directly uploaded to the Epi Info project to link with other variables in the database. Paper copies of field site questionnaires and all laboratory assay results are retained as backup.

### Data analysis plan

Statistical analyses will be done in SPSS 17.0 for Windows (SPSS Inc., Chicago, IL, USA). Data analysis will be conducted on intent-to-treat principles. Distributions of study variables will be analyzed to identify outliers and test assumptions of normality and equal variance. Data will be transformed to create normal distributions as needed. Randomization will be checked by evaluating infant sex, mode of delivery, gestational age, birth weight, mother age, BMI, and parity status using the *t*-test for continuous and the chi-square test for categorical variables. These variables will be used as covariates in multiple linear regression models to assess treatment effect on the continuous outcome variable of immune measures. If these adjustments have any considerable impact on the findings, the effect of the adjustment will be reported along with the effect without adjustment to evaluate whether the adjustment process interferes with the randomized comparisons. An interaction term, for example, treatment x postpartum time point of supplementation, will be used in mixed model multivariate analysis followed by inspection of univariate *F*-ratios. Interactions and main effects will be further analyzed for specific mean differences using *post hoc* analyses with a correction of alpha. Effect sizes associated with univariate *F*-statistics will be expressed as eta-squared (η^2^) for providing an indication of variance explained by the main effect or interaction. Effect sizes based on a specific difference in mean will be expressed as Cohen’s *d* for an indication of the magnitude of differences. Bivariate correlation in conjunction with multiple regression analysis will be used to evaluate inter-relationships among different immune measures associated with the effect of VAS.

## Discussion

During early life, an infant is not capable of producing immune regulatory factors sufficient for an adequate host defense. Breast milk bioactive compounds can compensate for the developmental delay of the recipient immune system by providing necessary support at the mucosal or systemic sites. We hypothesize that high dose VAS during the postpartum period would improve immune regulatory factors of breast milk and consequently improve infant immune status in geographical settings where the prevalence of vitamin A deficiency is very high. However, the concentration of vitamin A in breast milk is highest in the first 3 weeks postpartum, that is, in the colostrum in the first week and in the transitional milk in the next 2 to 3 weeks [46]. Following this period, well-nourished mothers usually have stable vitamin A concentrations during the lactation [47], while deficient mothers may have breast milk with lower concentrations [46]. However, if the mother cannot meet the increased vitamin A requirements during lactation through diet, her body attempts to compensate by drawing on the vitamin A reserves in the liver [48]. Thus, maternal dietary intake is an important determinant of breast milk vitamin A concentrations and an infant’s vitamin A status [46,49]. Considering this, we anticipate that data from Group 2 mothers in our study, who receive a single 200,000 IU vitamin A supplement at 6 weeks postpartum, may show higher breast milk immune components than data for Group 1 mothers, who receive the same dose supplement at <3 d postpartum. This prediction is supported by the observation that maternal supplementation with 400,000 IU within 24 hours postpartum does not improve breast milk status at 3 or 6 months [50]. However, a study that used a 300,000 IU dose at 1 to 3 weeks postpartum reported improved breast milk retinol concentrations at 6 to 9 months postpartum [51], indicating that a postpartum period of supplementation is important for breast milk retinol status. However, high dose VAS results in an immediate increase in circulating retinol concentration in plasma and breast milk that takes 3 to 4 weeks to equilibrate with liver storage. Considering this biological phenomenon, we predict that women in Group 3, who receive a second dose of vitamin A 6 weeks after the first dose (total: 2 x 200,000 IU), would have more sustained vitamin A stores and thus more breast milk immune components.

To our knowledge, this is the first placebo-controlled randomized clinical trial of postnatal VAS to investigate the key bioactive compounds in breast milk, important for infant immunity, in relation to dose and time point of postpartum supplementation and whether such maternal supplementation improves infant immune status during the critical period of early infancy when the infant experiences adaptation to both non-pathogenic and pathogenic organisms. Specifically, characterizing the adaptive immune responses in infants following the vaccination schedule, we will be able to evaluate the duration and nature of immune competence against infections in general. Time points for maternal supplementation are selected based on convenience. Mothers who visit health facilities or hospitals for delivery usually stay 3 to 4 days after normal delivery with longer stays for cesarean delivery. Another opportunity for the mothers to visit the clinic is 6 weeks postpartum in order to start immunization for their babies under the national infant immunization program. Our study results would help policymakers to consider postpartum VAS programs without introducing any additional visits by the mothers.

Our study does not aim to determine the impact of postpartum VAS on morbidity or mortality, which would require larger sample sizes and population-representative samples. Our prime goal was to undertake inclusive analysis of immune parameters in breast milk and infant blood samples, and for pragmatic reasons, we restricted our catchment area to one urban maternity clinic, from which blood samples could reach the icddr,b laboratory within 3 to 4 hours of collection. The prevalence of vitamin A deficiency among pregnant women in that area is known to be high and similar to that in rural areas in Bangladesh [52,53], but considering disease burden and environmental factors, urban and rural settings might differ. Enrollment at the maternity clinic rather than in the community did not skew our study subjects towards higher socio-economic status, since this public maternity clinic provides maternal and child care at highly subsidized rates or at no cost.

In summary, the ongoing study is an effort to understand the biological rationale for the potential effect of postpartum vitamin A supplementation with appropriate doses and postpartum time points to ensure adequate vitamin A and bioactive compound supply in breast milk to meet an infant’s optimum immune development as well as the daily vitamin A requirement.

### Trial status

This study has been in the active recruitment phase since April 2014. Anthropometry and analysis of infant’s fresh blood samples including CBC, mononuclear cell culture, and whole blood culture with TLR agonist and with mitogen PHA are being carried out. After the end of follow-up, laboratory analysis of stored frozen samples, for example, breast milk, plasma, culture supernatant, and mononuclear cells, will be performed in batches.

### Institutional Review Board (IRB)

Research Administration Services

International Centre for Diarrhoeal Disease Research, Bangladesh (icddr,b).

GPO Box 128

Dhaka 1000. Bangladesh

Phone: (+88 02) 9886498 or

PABX: (+88 02) 8860523–32 Ext: 3206

Fax: (+88 02) 9827075, 9827077

## Abbreviations

ALS: antibody in lymphocyte supernatant
BCG: bacillus Calmette-Guérin (vaccine)
CBC: complete blood count
ELISA: enzyme-linked immunosorbent assay
EPI: expanded program for immunization
FBS: fetal bovine serum
GM-CSF: granulocyte-monocyte colony-stimulating factor
HPLC: high-pressure liquid chromatography
IFN: interferon
IL: interleukin
IU: international unit
LPS: lipopolysaccharide
MCHTI: Maternal and Child Health Training Institute
ODN: oligodeoxynucleotides
OPV: oral poliovirus vaccine
PENTA: Pentavalent vaccines comprise diphtheria, tetanus, whole cell pertussis, hepatitis B, and *Haemophilus influenzae* type b conjugate vaccines
sCD14: soluble cluster of differentiation antigen 14
sIgA: secretory immunoglobulin A
TGF: transforming growth factor
Th: T helper cell
TLR: Toll-like receptor
TNF: tumor necrosis factor
VA: vitamin A
VAS: vitamin A supplementation
WHO: World Health Organization
